# Beta Diversity Patterns of Post-fire Forests in Central Yunnan Plateau, Southwest China: Disturbances Intensify the Priority Effect in the Community Assembly

**DOI:** 10.3389/fpls.2018.01000

**Published:** 2018-07-11

**Authors:** Jie Han, Zehao Shen, Yiying Li, Caifang Luo, Qian Xu, Kang Yang, Zhiming Zhang

**Affiliations:** ^1^Department of Ecology, College of Urban and Environmental Sciences, Peking University, Beijing, China; ^2^School of Ecology and Environmental Sciences, Yunnan University, Kunming, China

**Keywords:** community assembly, fire-prone ecosystem, species turnover, nestedness, spatial distance, topography, year-since-fire, soil nutrient

## Abstract

Post-fire succession is an ideal case for studying effects of disturbance on community assembly, and the key is to disentangle the contributions of assembly processes to the variation of community composition, namely beta diversity, and the contingent scales. The central Yunnan Plateau of Southwest China is characterized by monsoon related seasonal drought, and frequent forest fires. We sampled five fire sites burned in different years and a middle aged forest, measured species composition dissimilarity and its species turnover and nestedness components, within each fire site and across all sites. Results indicated species turnover as the primary component of beta diversity within all communities. There was no trend of change with year-since-fire (YSF) in beta diversity among early post-fire communities, but beta diversity in the middle aged community was significantly higher. Species turnover patterns across fire sites revealed a weak dispersal limit effect, which was stronger at lower than upper slope position for woody plants, and reverse for herbs. At the site scale, the species dissimilarity and turnover both enlarged with increasing slope position difference, especially in the middle-aged community, but the species nestedness had no consistent trend among sites, except a decreasing trend in the middle-aged forest. (Partial) Mantel tests indicated habitat filtering [primarily indicating total nitrogen (TN) and slope position] played a much stronger role than dispersal limit and YSF (indicating competition intensity) for the post-fire forest assembly at the landscape scale, for both woody and herbaceous layers. However, at the site scale, Mantel tests indicated a diminishing effect of soil nutrient filtering with increasing YSF, while effects of topography and spatial distance in the middle aged community was stronger. This divergence suggests the primary assembly mechanism gradually shift away from the soil constraint. While the seasonal drought and the mountain topography dominate the environmental legacy, our results imply that fires may reinforce a priority effect in the forests assembly in this region, by creating a habitat filtering (e.g., moisture and nitrogen limitation) effect on species composition in post-fire communities.

## Introduction

The secondary vegetation dynamics after disturbances, including wildfires, have long been a central theme of ecological succession studies (Clements, 1916; Watt, 1947). This large body of works has mostly focused on the direction and trajectory of community development, and the underlying drivers (West et al., 1981; Shugart, 1984). In the last decades, community studies have moved the emphasis to the changes of biodiversity and related ecosystem functions (Bengtsson et al., 2000; Franklin et al., 2002; Garnier et al., 2004), and the new emerging concept of community assembly provided an alternative perspective and analyzing models on the community processes, a fundamental topic in ecology (Gotzenberger et al., 2012).

Based on the niche differentiation of species, community assembly first refereed to the competition effect on species composition (Diamond, 1975). The effects of habitat filtering and the dispersal limitation on species pool were then included as critical community assembly processes (Keddy, 1992). In the last decade, a novel solution has been looking for to integrate the niche and neutral mechanisms for community assembly, and had stimulated a prominent wave of testing studies on the roles of related assembly processes (Kembel, 2009; Niu et al., 2009). Recently, this type of studies was criticized for using diversity patterns from a single (or short) timespan to infer past processes and mechanisms (Chang and HilleRisLambers, 2016). Applying community assembly models in the vegetation succession requires additional considerations across spatial and temporal scales (Kraft et al., 2011; Myers et al., 2013; Burkle et al., 2015). In contrast, the traditional succession theory placed the processes that influence community structure in an explicitly temporal context that was proposed by two competing models. One is the relay floristics model that describes a successive replacement of species groups. This model suggests that the dominance of one group creates conditions favorable for colonizing of a next group (Cowles, 1911; Clements, 1916; Connell and Slatyer, 1977). The other is the initial floristic composition model, which predicts that some species present in the early succession stage will persist through the succession process (Gleason, 1926; Egler, 1954). Although being controversial, the succession theories commonly took the historical information, such as the priority effect into considerations of community assembly (Zhang, 2014; Fukami, 2015). Recently, increasing efforts has emerged to integrate the theories of community assembly and succession, although a clear perspective is yet to be inferred from divergent results (Bruelheide et al., 2011; Raevel et al., 2012; Ulrich et al., 2016). Natural disturbances such as wildfires, by breaking the biotic and abiotic legacies and resetting the start point for community assembly processes, provide a unique opportunity in exploring effects of succession on assembly rules (Myers et al., 2013, 2015; Liu et al., 2016; Harms et al., 2017).

Being used to measure the variations of specie composition, the concept and indices of beta diversity have long been in the central of community assembly studies (Graham and Fine, 2008). No matter how was beta diversity defined (Baselga, 2010; Tuomisto, 2010), the spatial and temporal patterns of beta diversity provided useful information for disentangling the community assembly processes such as dispersal limitation, habitat filtering, and interspecific competition, and assessing their roles at various scales (Condit et al., 2002; Segre et al., 2014). For example, Myers et al. (2013) found that beta diversity in temperate and tropical forests reflects dissimilar mechanisms of community assembly. He also suggested that disturbance did not alter the relative importance of community assembly mechanisms (Myers et al., 2015). Liu et al. (2016) found environmental filtering is more important in community assembly of subtropical forests than spatial distance and disturbance legacy. Thus, comparison of beta diversity in a temporally and spatially explicit context could be expected to shed new light on the effect of disturbance on community assembly.

Wildfire is a prominent disturbance in many ecosystems. At a short temporal scale, fires act as an outside agent interrupting the post-fire community assembly, and set the starting point for succession (Turner et al., 1998); In the long run, the fire regime maintain a suit of heterogeneous habitats, and act as a selective driver to the evolution of functional traits for species and ecosystems (He et al., 2011; Pausas, 2014; Harms et al., 2017; Archibald et al., 2018). Moreover, fires create empty habitats by eliminating local populations accidentally and leave space for random dispersal and colonization (Freeman et al., 2007; Clair et al., 2016). Therefore wildfires can produce complicate, both deterministic and stochastic, effects on community assembly, resulting in divergent trajectories of community succession at local scale; and maintain a stable dynamics of metacommunity structure at landscape scale (Vanschoenwinkel et al., 2013). However, interactions between fires and community assembly rules, their relative roles and acting scales are still challenging issues in particular environmental and biotic legacies.

The central Yunnan Plateau in Southwest China possesses a sub-humid subtropical climate that is enormously influenced by the Indian Ocean Monsoon (Jiang, 1980), with a significant dry season generally last for months in the warm winter and spring. Meanwhile, the central Yunnan Plateau has experienced a long history of agricultural activities, which shaped the landscape into a mosaic of farmlands, settlements, secondary forests and shrubs (Jin and Peng, 1998; Guo et al., 1999). Partially due to the seasonal drought in climate, but mostly ignited by human activities, forest fires are widely observed and make this region a hotspot of forest fires in China (Zhang et al., 1994; Su et al., 2015; Ying et al., 2018). Sustainable management of this fire-prone ecosystem requires a solid understanding of changes in community composition and structure during the restoring process, and contributions of the deterministic and stochastic processes to the community assembly (Tempel et al., 2014; Boiffin et al., 2015). However, the role of fires in the assembly of the forest landscape mosaic, and in the succession trajectory of secondary forests have been scarcely studied in this region, thus interactions between fire disturbances and other community assembly processes is an open question yet to be answered.

Based on field investigation of the forest communities that were burned in different years, we detected the spatial patterns of beta diversity and its turnover and nestedness components of the post-fire communities in this region, in order to disentangle contributions of the deterministic and stochastic processes in the post-fire assembly, and elucidate the effect of fire disturbance on the assembly processes of post-fire forest communities under the regional environmental legacy. We specifically addressed the following questions: (1) What are differences between the spatial and temporal patterns of beta diversity and its nestedness and turnover components in the post-fire forest communities and a middle aged community? (2) What are differences of variation in beta diversity between the woody and herbaceous layers? (3) How much do the spatial distance, year-since-fire (YSF), and topography related environmental factors contribute to the community assembly of the post-fire forests in the central Yunnan Plateau?

## Materials and Methods

### Study Area

**FIGURE 1:**
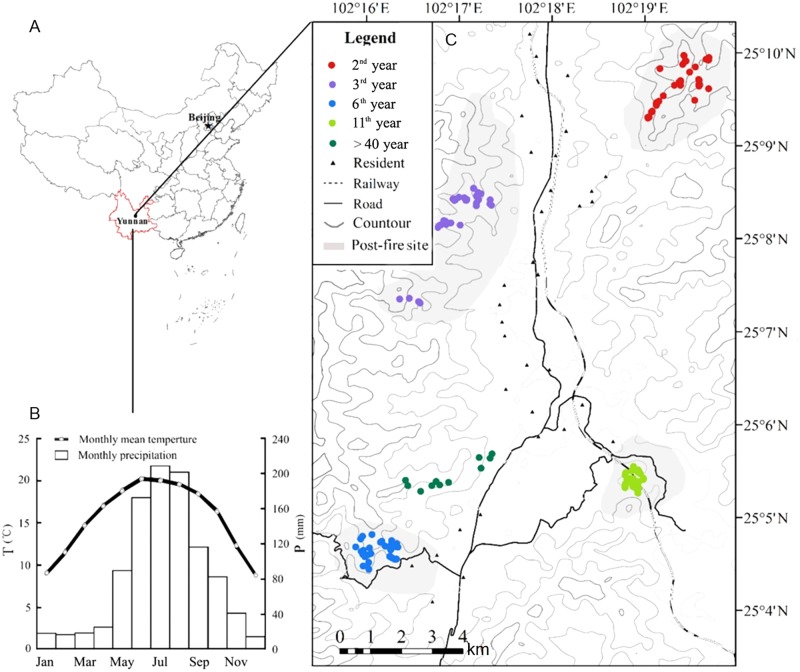
The geographical conditions of the forest fire sites and sampling points. **(A)** The geographical location of the study area; **(B)** the monthly precipitation and mean temperature of the Qinfeng meteorological station (25°9′2.75″N, 102°4′44″E; 1,650 m a.s.l.); **(C)** locations of fire sites and sampling points.

The study was performed in Qinfeng Town, a hilly region mixed with forests and paddy field patches in the central Yunnan Plateau, Southwest China. The elevation of this area ranges 1,780–2,615 m a.s.l. The climate is of subtropic monsoon type, the average annual temperature and precipitation are 14.6°C and 912 mm, respectively. The maximum and minimum monthly mean temperature are 32°C (July) and −2°C (January). The seasonality in precipitation is prominent, with 75% precipitation occurred from June to September (**Figure 1**). A dry season generally begins in November and ends by next May (Han et al., 2016).

The dominant vegetation in the study region is the secondary mixed forests of coniferous and broadleaved species. The dominant conifer species are Yunnan pine (*Pinus yunnanensis*) and shrubby Dipan pine (*P. yunnanensis* var. *pygmaea*), the dominant evergreen tree species include *Lithocarpus dealbatus*, *Cyclobalanopsis glaucoides*, *Michelia yunnanensis*, *Myrica nana*, while the dominant deciduous trees include *Lyonia villosa* and *Quercus acutissima*. Due to the rainfall seasonality and intensive human activity, the central Yunnan Plateau is a hotspot of forest fires (Zhang et al., 1994; Su et al., 2015). Repeated forest fires have led to widespread habitat degradation, as indicated by wide distribution of scrubs in the mountains, dominated by *P. yunnanensis* var. *pygmaea*, and the evergreen *Q. rehderiana* (Jin and Peng, 1998). In our study area, the typical mountain landscape of mixed forests was burned by four heavy fires in 2005, 2010, 2013, and 2014, respectively (**Figure 1**). The coverage of the four fires were in turn 290, 413, 1,240, and 235 ha, all were severe fires with the canopy mostly destroyed.

### Field Sampling of Plant Community and Habitat

From July to September in 2015, we surveyed the four fire fields and investigated the generation of post-fire forest communities. Since the monsoon general comes in early June, forest fires in this region all occurred from January to May, and the post-fire regeneration well begins during the contemporary growing season. Therefore, communities in the four fire site have experienced 2, 3, 6, and 11 years post-fire regeneration (**Supplementary Figure S1**). Within the study region, we also select an area of middle-aged forest that has not been burned in last 40 years (based on visiting to local people). We investigated a group of forest stands within this area, as a baseline condition for comparing with the burned stands.

With all merits and pitfalls, the space-for-time approach has been widely applied in ecological studies (Kappes et al., 2010; Thomaz et al., 2012). Although no repetition of fires was recorded in each of 2, 3, 6, 11 years before this study, these fire sites can be reasonably viewed as repetitions of a sample of early-staged post-fire communities, with regard to the life span of forests in this region. And 36 forest plots that were not burned in last 40 years can be viewed as a control in comparison, as a middle-aged sample of forest in terms of the species composition and population structure.

We investigated communities on four post-fire sites and the controlling site following a stratified random design. At each site, we select a typical topographic profile in south to north direction, and set our sampling plots at five slope positions: valley bottom, lower side slope, middle slope, upper side slope (or saddle), and hilltop (**Supplementary Figure S1**). At each topographic position, we randomly selected four plots of 20 × 20 m^2^ area for plant community and habitat investigation. Thus we sampled 4 × (2 × 4 + 1) = 36 plots on each burned site. The following analyses were based on data of all 180 plots of forest community with different years of post-fire regeneration.

The content of plot investigation included geographical location, habitat conditions, including topography and soil features, survival trees and dead poles of pre-fire canopy, and post-fire regeneration (**Table 1**). Geographical coordinates included latitude, longitude and elevation. Topographic features include slope exposure (from north = 0 to south = 180), slope inclination, slope position (**Supplementary Figure S2**), and horizontal slope shape (convex, straight, and concave). We then quantified the topographical features following Shen et al. (2000). We investigated plant community structure of both woody and herbaceous layers in each plot. For woody plants, we measured and recorded the species names, regeneration form (seedling or sprout), stem height (m), diameter at breast height (DBH, cm) of each individuals. For sprouting plants in particular, the number of sprouts and the coverage were also counted and visually measured. For herbaceous plants, we recorded the name, abundance class apply in Drude’s seven classes regime (Jin and Peng, 1998), and percent coverage of each species. In each plot, we randomly selected three points to sample surface soil. At each point, we removed the litter and humus layer and collected 500 g top soil at the depth of 0–10 cm, then we thoroughly mixed three soil samples in the field and took a random sub-sample of 500 g.

**Table 1:** Type and value range of habitat variables of the burned sites in this study.

Factors	Variable	Abbr.	Value range and notes
Coordinates	Latitude, longitude	Lati, long	Decimal value
	Elevation (m)	Elevation	1,800 ~ 2,300
Topography	Slope inclination (°)	Slope	2 ~ 55
	Slope aspect	Aspect	Arccosine transformed to 0 (N) ~ 1(S)
	Slope shape	Shape	Concave-1, plain-2, convex-3
	Slope position	Position	Hilltop-5, ridge-4, midslope-3, foot-2, valley bottom-1
Soil	pH value	pH	3.57 ~ 6.11
	Sand (%)	Sand	0 ~ 100
	Total nitrogen (%)	TN	0.74 ~ 1.23
	Total carbon (%)	TC	0.96 ~ 16.07
	Organic Carbon (%)	OC	0.94 ~ 16.04
	Inorganic carbon (%)	IOC	0 ~ 0.53
	Available phosphorus (%)	P	0 ~ 0.02

### Soil Analyses

A total of 180 fresh soil samples were placed into pre-labeled plastic bags and shipped back to the Biogeochemistry Laboratory in Peking University. In the laboratory, each soil sample was adequately mixed and divided into four equal sub-samples, one sub-sample was used for measuring pH values as immediately as possible, using a potentiometer (S20P-K) in fresh soil after water extraction (soil : water was 1:2.5). The other sub-samples were air-dried, smashed and sieved using 2 mm mesh. A 50 g sub-sample was stored separately for the experiment of soil text, and the rest soil was sieved using 0.15 mm mesh and stored in plastic bags for total C, total N, inorganic C, and extractable P analysis.

The soil texture analysis of a 50.0 g sub-samples was pre-proceeded with H_2_O_2_ and HCl to neutral in pH value, then the soil particle size was measured using the Malvern Laser Particle Size Analyzer (MS2000). For the soil extractable P, a sub-sample of 5.00 g soil was prepared and measured using the Molybdenum Blue Colorimetric Method. The total C and total N was measured with the Dry Combustion Method using the Elemental Analyzer (Model: variomacro cube, Germany), and the inorganic C was measured with the Gasometric Method using a Carbonate Analyzer (Eijkelkamp 08.53).

### Statistical Analyses

#### Beta Diversity Indices

As an indicator of variation in species composition among communities, the Sorenson index of beta diversity measures two basic community processes, i.e., species gain or loss (via nestedness) and species replacement (turnover) (Baselga, 2010). Thus, β_Sor_ = β_nestedness_ + β_turnover_

Species nestedness describes the regional species composition when the species assemblage of a region is a subset of species assemblage of another region, reflecting non-random species loss caused by the decomposition of regional species assemblage by some factors.

Species turnover indicates that some species of a region are replaced by other species, under a particular environmental gradient, or spatial and historical constraints, indicating the effects of environmental filters. The functions for these factors are:

$$\beta_{\text{sor}}=\frac{\max(b,\;c)+\min(b,\;c)}{2a+\min(b,\;c)+\max(b,\;c)}$$

$$\beta_{\text{nestedness}}=\frac{\max(b,\;c)-\min(b,\;c)}{2a+\min(b,\;c)+\max(b,\;c)}\times \frac{a}{a+\min(b,\;c)}$$

$$\beta_{\text{turnover}}=\frac{\min(b,\;c)}{a+\min(b,\;c)}$$

where *a* is the number of species shared by two plots, *b* and *c* are number of species in two different plots. Larger values indicate higher community beta diversity. Beta diversity indices are calculated using the “betapart” package in R statistic software^1^.

#### Turkey–Kramer Test for Multiple Comparison

To test the difference of among communities of different YSFs, we applied the Tukey–Kramer test for multiple comparisons of beta diversity indexes, including Sorenson index and its nestedness turnover components. The Turkey–Kramer test allows for unequal sample sizes and provides test for each pair of multiple groups based on one-way ANOVA.

#### Mantel Test and Partial Mantel Test of Correlation Between Distance Matrixes

To decompose the effects of spatial distance and environmental differences on the variation of post-fire community assembly, we used the Mantel test and to estimate the matrix correlation of the species composition dissimilarity with the spatial distance, temporal distance, and the environmental distance, respectively (Legendre and Legendre, 1998). In addition, we then used the partial Mantel test to do variation partitioning for the independent contribution of each factor to the community assembly, with that of other two factors in control (Legendre, 2000). We constructed the geographic distance matrix by calculating the geographic distance between each pair of plots using the coordinates of each plot. The temporal distance matrix was constructed by the Euclidian distance between the year of burning in each plot, and the environmental distance matrix was constructed, for all environmental variables or specifically for a factor, also by the Euclidean distance between each pair of plots.

All analyses were implemented with corresponding packages in Software R 3.4.3. (R Core Team, 2011).

## Results

### Temporal Variation of Beta Diversity of Post-fire Communities

**FIGURE 2:**
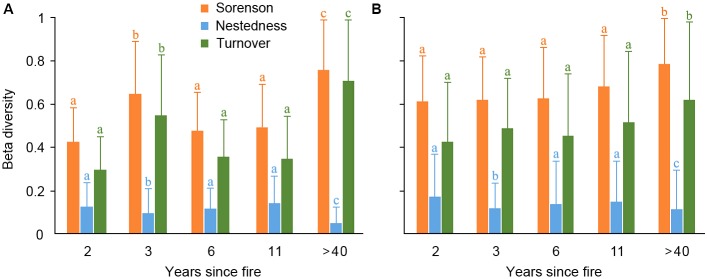
Total Beta diversity (Sorenson index, yellow) and its nestedness (blue) and turnover (green) components in the woody layer **(A)** and herbaceous layer **(B)** of post-fire plant communities of different year-since-fire (YSF). The error bar indicates the standard deviation. The letters on top of the error bars indicated a Tukey-Kramer test for multiple comparisons among post-fire communities of different YSFs, which was applied specifically for each beta diversity index (indicated by the same color).

For woody species, the overall beta diversity (Sorenson index) was primarily composed of the turnover component in communities of different YSFs, and the nestedness component played a minor role (**Figure 2A**). The Sorenson index of controlling communities has significantly higher values than that of communities burned in recent years (*p* < 0.05), as revealed by a Tukey-Kramer test, while value differences of Sorenson index among recently burned communities were not significant, although the smallest value was in the 2-years post-fire communities. The turnover component revealed a similar variation as overall beta diversity in all communities.

For herb species, the temporal variation of beta diversity showed similar patterns as the woody species among communities of different post-fire years (**Figure 2B**). The controlling communities have the highest beta diversity, and turnover index was the major component of beta diversity. On the other hand, the beta diversity was not significantly different among communities burned in recent different years.

### The Relationship Between Beta Diversity and the Spatial Distance

**FIGURE 3:**
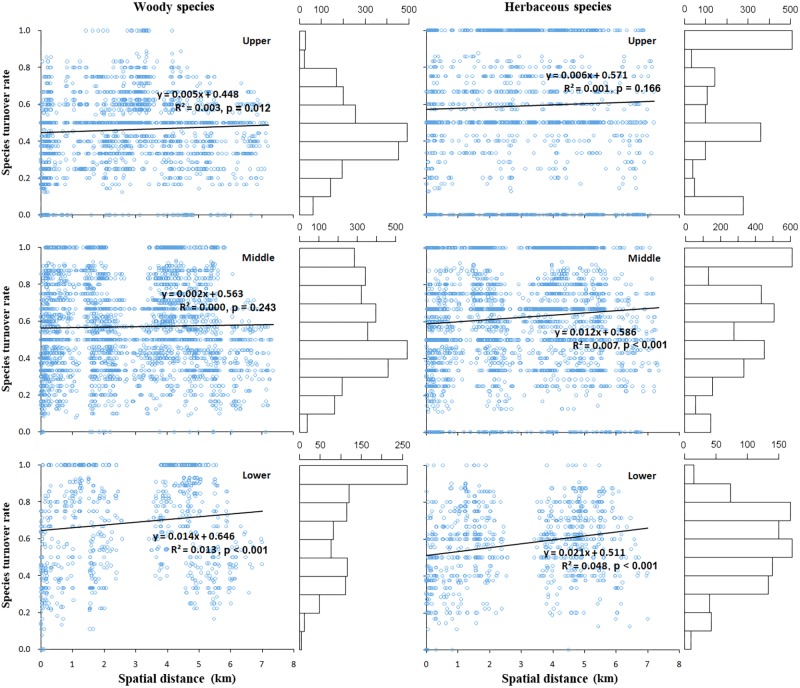
The correlation between species turnover rate and the Euclidian distance between pairs of plots, estimated separately for plots lying at lower, middle, and upper slope positions, and for woody (left) and herbaceous (right) plant species, respectively. The horizontal bar plot on the right side of each scatter diagram indicates the value frequency distribution of species turnover rate.

In general, the species turnover of post-fire plant communities (β_turnover_) was found to have a weak positive correlation with spatial distance between the communities (*p* > 0.05), for either the woody or the herbaceous layer, this correlation kept consistent at three slope positions (**Figure 3**).

Moreover, the species turnover increased with a generally higher rate in the herb than the woody plant layer, indicated by the regression coefficients at the upper (0.006 vs. 0.005), middle (0.012 vs. 0.002), lower (0.021 vs. 0.014) slope position, respectively; this pattern also indicated a larger species turnover rate presented at the lower than the upper position. Specifically, for the woody layer, the frequency of high specie turnover value (0.80–1.00) obviously decreased from lower to upper slope position, while the middle turnover value (0.40–0.50) prominently increased in contrast. For the herb layer, there was a high frequency of large turnover (0.60–0.80) at the lower position, whereas at the middle and upper slope position, the largest frequency of species turnover value occurred at the maximum rates (0.90–1.00).

### Topographic Patterns of Beta Diversity in Post-fire Communities

**FIGURE 4:**
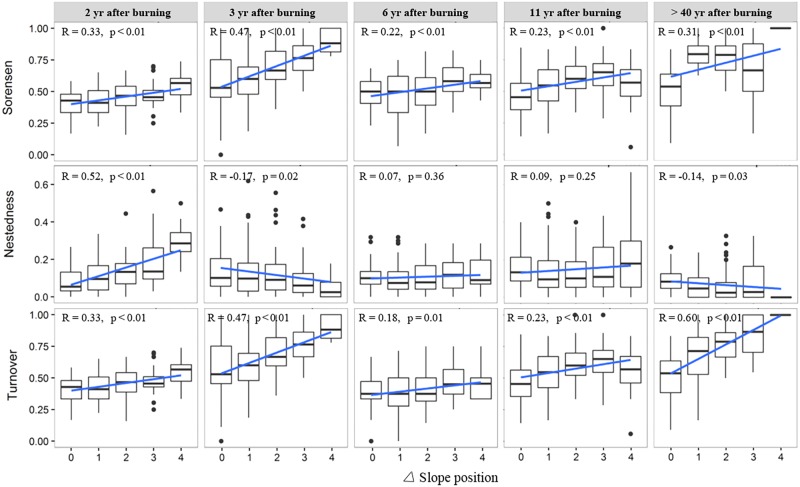
Change of beta diversity and its nestedness and turnover components in the woody layer of post-fire communities along with difference of slope position. R is the Pearson correlation coefficient between beta diversity indices and the slope position difference; *p*-values indicate the level of significance. Slope position indicate the slope position difference of each pair of plots.

The variation trend of species composition dissimilarity (Sorenson index) across the topographic positions, from valley bottom to ridge, was basically consistent for the woody layer of the post-fire regenerating communities of different YSFs (**Figure 4**). In all post-fire communities, the overall beta diversity increased significantly with increasing difference of slope positions (DSP). The species turnover always constituted the major component of beta diversity, and generally increased in value with increasing DSP between communities. On the other hand, the change of nestedness did not show a consistent trend with increasing DSP across post-fire communities of different YSFs. However, for the middle-aged communities (>40 YSFs), the increase of beta diversity and its turnover component, and the decrease of the nestedness component along with the increase of DSP were all statistically significant.

**FIGURE 5:**
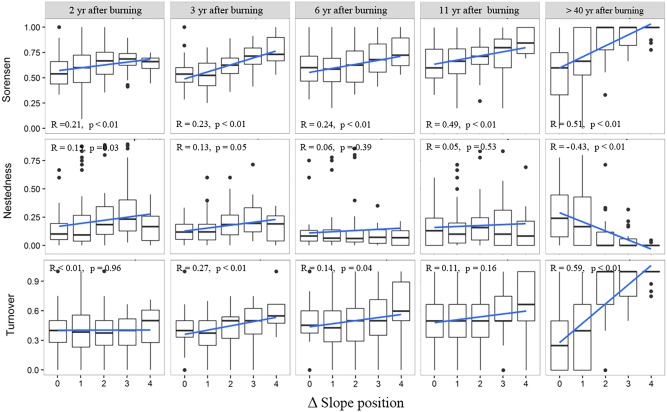
Changes of beta diversity and the nestedness and turnover components in the herbaceous layer of post-fire communities along with difference of slope position. R is the Pearson correlation coefficient between beta diversity indices and the slope position difference; *p*-values indicate the level of significance. Slope position indicate the slope position difference of each pair of plots.

The overall patterns of beta diversity of herbaceous layers of post-fire communities (**Figure 5**), along the gradient of DSP, were similar to those of the woody layer. With increasing DSP, the increase of species composition dissimilarity was significant, so was the increase of species turnover. However, the change of specie nestedness, as a minor component of beta diversity, did not show consistent pattern along with increasing DSP in recently burned regenerating communities, but in the middle-aged communities (>40 YSFs), the nestedness significantly decreased with an increasing DSP.

### Determinants of Spatiotemporal Patterns of Beta Diversity for Post-fire Communities

**FIGURE 6:**
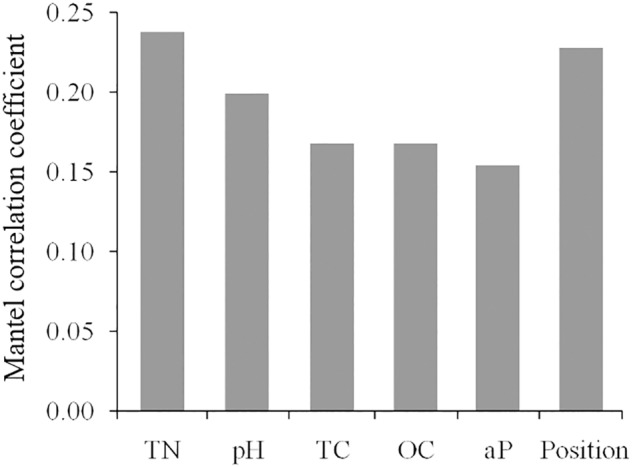
Coefficients of significant correlation between the distance matrix of environmental variables and the beta diversity matrix. TC, total carbon; TN, total nitrogen, OC, organic carbon; aP, available phosphorus; Position, slope position.

For all the 180 plots of post-fire communities, the Mantel test revealed significant and positive correlations between beta diversity the temporal distance, the spatial distance and the environmental distance. Specifically, the distance of soil factors had a higher correlation than that of topography with the beta diversity (**Table 2**). For all measured topographic and soil variables, the total nitrogen (TN) was a best single predictor (*R* = 0.237) for the variation of beta diversities, followed by the slope position (*R* = 0.228; **Figure 6**). Moreover, the partial Mantel test suggested that the independent effect of environmental distance on species composition dissimilarity is obviously stronger than that of the temporal or spatial distances.

**Table 2:** Mantel test and partial Mantel test for the effect of temporal, spatial distance on beta diversity ~ environmental distance correlation in post-fire communities.

YSF	n	Spa	Temp	Env	Topo	Soil	Env| Spa	Spa| Env	Env| Temp
2	36	0.165^∗^	–	0.340^∗∗^	0.294^∗∗^	0.157^∗∗^	0.320^∗∗^	0.114^∗^	–
3	36	0.266^∗∗^	–	0.523^∗∗^	0.449^∗∗^	0.378^∗∗^	0.498^∗∗^	0.195^∗^	–
6	36	0.084	–	0.195^∗^	0.137^∗^	0.122^∗^	0.193^∗^	0.080	–
11	36	0.165^∗^	–	0.194^∗^	0.134^∗^	0.101^∗^	0.183^∗^	0.152^∗^	–
>40	36	0.286^∗∗^	–	0.230^∗^	0.311^∗∗^	0.084	0.129^∗^	0.233^∗^	–
Total	180	0.052^∗^	0.111^∗∗^	0.284^∗∗^	0.177^∗∗^	0.237^∗∗^	0.282^∗∗^	0.045	0.278^∗∗^

*Env, Environment = Topo + Soil; Spa, Spatial; Temp, Temporal; Topo, Topography. Level of significance: ^∗^p < 0.05; ^∗∗^p < 0.01; ^∗∗∗^, p < 0.001*

Compare the causal factors at the fire site scale, the matrix correlation coefficients between community beta diversity and the environmental distances (*R* = 0.194 ~ 0.523) were consistently higher than that for the spatial distances (*R* = 0.084 ~ 0.286), the importance of environmental difference over spatial distance for beta diversity were also confirmed by the Env| Spa vs. Env| Spa values in all sites of different YSFs.

In addition, soil distance was more important than topographic distance for beta diversity of plant communities across the study area, which is composed multiple fire sites and un-burned area. However, within the scale of specific fire site, the topography distance was consistently more important than soil distance on beta diversity among communities of different YSFs. Moreover, the independent effect of environmental distance (Env| Spa) seemed to decline along with increasing time of forest restoration, the effect of soil also followed a similar trend. In contrast, the effect of spatial distance and topographic difference on beta diversity recovered in the middle- aged forests, compared with the early post-fire plots.

## Discussion

### The Initial and Relay Floristic Components of Post-fire Regenerating Communities

For the within-site variation of species composition, there wasn’t an obvious trend of species turnover change in the regeneration of forest communities over the first 10 years. This was indicated by the insignificant difference of the mean beta diversity and its two components, species turnover and nestedness, among the 2, 6, 11-years post-fire communities (**Figure 3**). In other words, the species composition of each fire site was homogenized by the disturbance, and this is also indicated by the significantly higher beta diversity in the 40 years aged forests, especially its turnover component. Han et al. (2015) compared the species compositions of pre-fire forests and the post-fire regenerations in this region, and found the *Jaccard* similarity reached 0.532 ± 0.220 for woody layer after only one growing season, with population density taken into account, and the species composition similarity in the herb layer also reached >0.5 in the following year (Han et al., 2016). In general, post-fire forests of this region recovered very rapidly with abundant regeneration. This process should be facilitated by the ample rainfall in the summer and autumn (>700 mm), and fire-adapted regenerative strategies, such as serotiny and resprouting that are commonly possessed by woody species in this region (Su et al., 2015). Therefore, both the flora and structure of the post-fire communities are composed of the “initial” and “relay” components (Kayes et al., 2010; Donato et al., 2012): a persistent “initial” component that is very resilient to fire disturbances, recovered actively in the same year, with the help of fire-adapted regenerating strategies and favorable post-fire climate condition; this floristic component dominate the early stage of the post-fire communities. A “relay” component seems to be more sensitive and vulnerable to fire disturbance, will come back later to take the place in the middle-aged forests in this region, but the process was not obvious at least in the early 10 years after fires.

### Differential Patterns in Beta Diversity Components and Community Layers

Baselga (2010); Gotzenberger et al. (2012) decomposed beta diversity into the turnover and the nestedness components. The turnover reflects species replacement along an environmental gradient, and the nestedness indicates species loss driven by local population extinction and new species invasions. The species replacement that caused by distribution limit is generally related to the geographical distance and/or environmental gradient in a heterogeneous landscape, namely dispersal limit or habitat filtering (Hardy and Sonke, 2004; Franklin et al., 2013; Cao et al., 2016; Dambros et al., 2017). At the site scale in this study, spatial distances between sampling plots of neighboring topographic positions were less than 1 km, however the species turnover generally constituted a major part in beta diversity of the post-fire regenerating communities (>70%) compared with the nestedness component, this value was even larger (>80%) in both woody and herb layers of the middle-aged forest communities (**Figure 2**). Such a composition of beta diversity suggested the environment gradient as a primary driver of community assembly in the post-fire restoration. At a landscape scale (**Figure 1**), the species turnover of all studied communities only revealed a very weak (if any) correlation with the spatial distance, and the correlation also varied with topography (**Figure 3**), generally more significant at the lower slope position. The correlation was stronger for the herb than the woody laye. This pattern suggested the role of dispersal limit was also weak for the assembly of metacommunity at the landscape scale, although larger for the distribution of herb species than woody species. In addition, a significant signal of topographic effect was also revealed in the spatial effect, which had a larger effect to species dwelling at the lower slope position (i.e., valley bottom) than to those prevalent at the upper slope position, where the communities are more frequently and severely disturbed by fires, as indicated by many fire studies (Keeton and Franklin, 2004; Lentile et al., 2006; Wu et al., 2013).

### Deterministic and Stochastic Components in Post-fire Community Assembly

Wildfires generally cause local population extinction, create empty space for invaders, and initiate a secondary community succession in post-fire habitats (Scott et al., 2014). The contributions of the “initial” and “relay” floristic components, or the persistent and invading species, in the community assembly is a combination of deterministic mechanisms including environmental filtering, biological interaction and population growth, as well as the dispersal limit as a stochastic driver (Leibold et al., 2004). In spite of numerous studies addressing relative contributions of these mechanisms in shaping beta diversity patterns of post-fire communities, existing results have been divergent and sometimes conflicting (Gotelli and Mccabe, 2002; Murphy et al., 2014; Shryock et al., 2015; Catano et al., 2017). The key underlying the controversy was probably the diversity of historical legacies that were region-specific combinations of interactive environmental and stochastic factors (Brown et al., 2015; Basnou et al., 2016; Conradi et al., 2017), such as the monsoon-driven climate, the hilly topography on the plateau, and the mosaic of secondary mixed forests in this studied region. Therefore, integrating multiple mechanisms, determinative and stochastic, into a scale-related hierarchical framework such as the metacommunity paradigm should be helpful for an appropriate understanding of the post-fire community assembly (Leibold et al., 2004; O’Dwyer et al., 2009; Lopez et al., 2016).

In our case, the environmental distance revealed obviously a larger contribution to beta diversity patterns than the temporal or spatial distances (**Table 2**). However, environmental factors seemed to work in a scale related framework. In the temporal dimension, since fires in the study region occurred mostly from January to May (Zhang et al., 1994), and the Indian Ocean Monsoon typically starts here in June, 2 months later than the onset of the growing season. Thus the post-fire regeneration is largely regulated by the precipitation in the same year of burning. The initial floristic components start a quick population recovery with the help of fire adapted regenerating traits, such as resprouting, serotiny and smoke-stimulated germination (Su et al., 2015, 2017; Huang et al., 2016), showing a priority effect on community assembly. Meanwhile, populations of other species invade from outside fire sites as a random process; also play some role in the early community assembly. For example, *Eupatorium adenophora*, a notorious invasive species was very rare and sparse in the forest of over 40 years ages, had achieved a considerable coverage on bare patches in the recently burned sites, much more abundant at the upper slope positions where the burning was generally severer (Han et al., 2016). On the other hand, in the middle aged community, slope position difference had a significant positive correlation with species turnover, and a negative correlation with nestedness. These relationships were especially prominent when the difference of slope position level was larger than 2, highlighting the contrast between the lower and the upper parts of a vertical slope profile (**Figures 4**, **5**). However, changes of beta diversity with slope position difference were much weaker and inconsistent among four communities at the early post-fire stage, in both the woody and herb layers, indicating a larger role of stochasticity in the early stage of post-fire succession, and an increase of regulating role by deterministic factors in the middle-aged communities. Specifically, this transition should related more to the “relay” rather than the “initial” floristic component, as also observed in the post-fire regeneration of other ecosystems (Måren et al., 2018).

In the spatial dimension, the environmental heterogeneity within each fire site was dominated by topographic features, slope direction for solar radiation, slope steepness and position for soil moisture and nutrient contents (Wu et al., 2013; Shryock et al., 2015). The plant invasion of habitat emptied by fires is also inevitably limited by the dispersal distance, especially for the “relay” components, although the constraint maybe not stronger than environmental filters, as suggested in **Table 2**. At a landscape scale cross the fire sites, however, the topography features such as slope direction and position should repeat spatially, both in the burned or unburned areas, and were thus less representative to habitat condition than soil features (such as TN) at a specific location, as indicated in **Table 2**. Moreover, the effect of spatial distance on beta diversity pattern was less important across all studied sites than within each fire site. Why? In a landscape mosaic of fire-prone forest that composed of post-fire patches of different YSFs, the meta- community assembly may not rely much on the long-distance species dispersal. Indeed, the mountain tops are more spatially isolated, and the forests are generally more severely damaged by forest fires, thus local population extinction should occur frequently and dispersal limit should play a larger role at the upper than lower slope positions, in contrast to the observed fact as indicated by the species turnover patterns (**Figure 3**). The only reason should be that, the restoration of the post-fire communities, especially habitat at the upper slope position, mostly rely on the initial (rather than rely) floristic components. The role of initial floristic components in post-fire community assembly revealed a priority effect that was not only determined by the regional environmental context, but also reinforced by the effects of persistent fire regime, through the general adaptation to fire disturbance in tree species regeneration strategies. In a comparative study of subtropical forests assembly, Liu et al. (2016) also found environmental filtering is more important than spatial distance in disturbed communities.

Among the deterministic factors, TN was found more relevant than other edaphic factors as the best single predictor to the beta diversity of plant communities. Since fires destroy above-ground biomass and burn humus in surface soil, soil erosion and nutrient leaching are also intensified. Increase of carbon and substantial loss of available nitrogen in soils turn organic N into a limiting factor for post-fire regeneration (Collier and Mallik, 2010; Pellegrini, 2016). In the studied fire sites, the effects of soil on the beta diversity of plant communities showed a decreasing trend with increasing YSF, while the effects of topography and spatial distance increased in the middle-aged communities (**Table 2**). This contrast probably implied that, along with the post-fire establishment of forest canopy and the nutrient accumulation in soil, the critical environmental filter gradually shifted from soil nutrients to other topography related factors, such as solar radiation and soil moisture.

Studying community assembly requires observations into related processes at multiple scales, this is especially important for the space-for-time approach. Limited by the number of fire sites sampled in this study, the effect of YSF on species composition is not adequately estimated. For better understanding the underlying mechanisms, sampling on more sites and long-term monitoring are both required for communities with different YSFs, so as to harbor the spatiotemporal variations of environmental legacy, and explore interactions of biotic and abiotic processes in post-fire forest dynamics. Nevertheless, our results reveal the significant difference in species composition between the early post-fire forests and restored communities, and the importance of soil nitrogen for the early stage of post-fire forest regeneration. This information could be useful for post-fire habitat conservation and forest fire management.

## Conclusion

The spatiotemporal patterns of beta diversity in the studied area shed new light on the assembly rules of post-fire forest assembly. Forest community composition did not show a progressive change in the early stage of post-fire regeneration, although was significantly different from the restored community. Topography related habitat filtering is more important than dispersal limit in the post-fire community assembly. During the post-fire restoration, the environmental limiting factor for plant regeneration shifts from soil N to other resource that also regulated by topography. By driving the evolution of fire-adapted regeneration strategies, the repeated fire disturbances strengthen the priority effects determined by the environmental legacy and the initial floristics. For the first time, this study highlighted the critical link between fire disturbance and regional environmental legacy in determining the post-fire forest species composition in the central Yunnan Plateau. Further studies are required to disentangle the interactions between priority effects and the post-fire forest assembly within a metacommunity framework.

## Author Contributions

ZS conceived and designed the study, and ZS wrote the manuscript. JH, CL, YL, QX, KY, ZZ, and ZS conducted the data collection. JH performed the experiments. JH and ZS analyzed the data.

## Conflict of Interest Statement

The authors declare that the research was conducted in the absence of any commercial or financial relationships that could be construed as a potential conflict of interest.

## Footnotes

1 https://www.r-project.org/

## Supplementary Material

FIGURE S1 The appearance of post-fire regeneration forests of different year-since-fires.

FIGURE S2 A diagram indicating slope positions of a vertical topographic profile.

## References

[B1] Archibald S. Lehmann C. E. R. Belcher C. M. Bond W. J. Bradstock R. A. Daniau A. L. (2018). Biological and geophysical feedbacks with fire in the Earth system. *Environ. Res. Lett.* 13 1–19. 10.1088/1748-9326/aa9ead

[B2] Baselga A. (2010). Partitioning the turnover and nestedness components of beta diversity. *Glob. Ecol. Biogeogr.* 19 134–143. 10.1111/j.1466-8238.2009.00490.x

[B3] Baselga A. (2012). The relationship between species replacement, dissimilarity derived from nestedness, and nestedness. *Glob. Ecol. Biogeogr.* 19 134–143. 10.1111/j.1466-8238.2009.00490.x

[B4] Basnou C. Vicente P. Espelta J. M. Pino J. (2016). Of niche differentiation, dispersal ability and historical legacies: what drives woody community assembly in recent Mediterranean forests? *Oikos* 125 107–116. 10.1111/oik.02534

[B5] Bengtsson J. Nilsson S. G. Franc A. Menozzi P. (2000). Biodiversity, disturbances, ecosystem function and management of European forests. *For. Ecol. Manage.* 132 39–50. 10.1016/S0378-1127(00)00378-9

[B6] Boiffin J. Aubin I. Munson A. D. (2015). Ecological controls on post-fire vegetation assembly at multiple spatial scales in eastern North American boreal forests. *J. Veg. Sci.* 26 360–372. 10.1111/jvs.12245

[B7] Brown C. D. Liu J. Yan G. Johnstone J. F. (2015). Disentangling legacy effects from environmental filters of postfire assembly of boreal tree assemblages. *Ecology* 96 3023–3032. 10.1890/14-2302.1 27070021

[B8] Bruelheide H. Böhnke M. Both S. Fang T. Assmann T. Baruffol M. (2011). Community assembly during secondary forest succession in a Chinese subtropical forest. *Ecol. Monogr.* 81 25–41. 10.1890/09-2172.1 25303438

[B9] Burkle L. A. Myers J. A. Belote R. T. (2015). Wildfire disturbance and productivity as drivers of plant species diversity across spatial scales. *Ecosphere* 6:202 10.1890/ES15-00438.1

[B10] Cao P. Wang J. Hu H. Zheng Y. Ge Y. Shen J. (2016). Environmental filtering process has more important roles than dispersal limitation in shaping large-scale prokaryotic beta diversity patterns of grassland soils. *Microb. Ecol.* 72 221–230. 10.1007/s00248-016-0762-4 27072664

[B11] Catano C. P. Dickson T. L. Myers J. A. (2017). Dispersal and neutral sampling mediate contingent effects of disturbance on plant beta-diversity: a meta-analysis. *Ecol. Lett.* 20 347–356. 10.1111/ele.12733 28093844

[B12] Chang C. HilleRisLambers J. (2016). Integrating succession and community assembly perspectives. *F1000Res.* 5:F1000 Faculty Rev-2294. 27785355 10.12688/f1000research.8973.1 PMC5022705

[B13] Clair S. B. O’Connor R. Gill R. McMillan B. (2016). Biotic resistance and disturbance: rodent consumers regulate post-fire plant invasions and increase plant community diversity. *Ecology* 97 1700–1711. 10.1002/ecy.1391 27859155

[B14] Clements F. E. (1916). *Plant Succession: An Analysis of The Development of Vegetation* Vol. 242 Washington, DC: Carnegie Institute of Washington Publication, 1–512. 10.5962/bhl.title.56234

[B15] Collier L. C. Mallik A. U. (2010). Does post-fire abiotic habitat filtering create divergent plant communities in black spruce forests of eastern Canada? *Oecologia* 164 465–477. 10.1007/s00442-010-1642-0 20461414

[B16] Condit R. Pitman N. Leigh E. G. Jr. Chave J. Terborgh J. Foster R. B. (2002). Beta-diversity in tropical forest trees. *Science* 295 666–669. 10.1126/science.1066854 11809969

[B17] Connell J. H. Slatyer R. O. (1977). Mechanisms of succession in natural communities and their role in community stability and organization. *Am. Nat.* 111 1119–1144. 10.1086/283241

[B18] Conradi T. Temperton V. M. Kollmann J. (2017). Beta diversity of plant species in human- transformed landscapes: control of community assembly by regional productivity and historical connectivity. *Perspect. Plant Ecol. Evol. Syst.* 24 1–10. 10.1016/j.ppees.2016.10.001

[B19] Cowles H. C. (1911). The causes of vegetative cycles. *Bot. Gaz.* 51 161–183. 10.1086/330472

[B20] Dambros C. S. Morais J. W. Azevedo R. A. Gotelli N. J. (2017). Isolation by distance, not rivers, control the distribution of termite species in the Amazonian rain forest. *Ecography* 40 1242–1250. 10.1111/ecog.02663

[B21] Diamond J. M. (1975). “Assembly of species communities,” in *Ecology and Evolution of Communities*, eds Cody M. L. Diamond J. M. (Cambridge, MA: Harvard University Press), 342–444.

[B22] Donato D. C. Campbell J. L. Franklin J. F. (2012). Multiple successional pathways and precocity in forest development: can some forests be born complex? *J. Veg. Sci.* 23 576–584. 10.1111/j.1654-1103.2011.01362.x

[B23] Egler F. E. (1954). Vegetation science concepts I. Initial floristic composition, a factor in old-field vegetation development. *Plant Ecol.* 4 412–417. 10.1007/BF00275587

[B24] Franklin J. F. Spies T. A. van Pelt R. Carey A. B. Thornburgh D. A. Berg D. R. (2002). Disturbances and structural development of natural forest ecosystems with silvicultural implications, using douglas-fir forests as an example. *For. Ecol. Manage.* 155 399–423. 10.1016/S0378-1127(01)00575-8

[B25] Franklin J. Keppel G. Webb E. L. Drake D. R. (2013). Dispersal limitation, speciation, environmental filtering and niche differentiation influence forest tree communities in West Polynesia. *J. Biogeogr.* 40 988–999. 10.2307/23463615

[B26] Freeman J. P. Stohlgren T. J. Hunter M. E. Omi P. N. Martinson E. J. Chong G. W. (2007). Rapid assessment of postfire plant invasions in coniferous forests of the western United States. *Ecol. Appl.* 17 1656–1665. 10.1890/06-1859.1 17913130

[B27] Fukami T. (2015). Historical contingency in community assembly: integrating niches, species pools, and priority effects. *Annu. Rev. Ecol. Evol. Syst.* 46 1–23. 10.1146/annurev-ecolsys-110411-160340

[B28] Garnier E. Cortez J. Navas M. L. Roumet C. Debussche M. Laurent G. R. (2004). Plant functional markers capture ecosystem properties during secondary succession. *Ecology* 85 2630–2637. 10.1890/03-0799

[B29] Gleason H. A. (1926). The individualistic concept of the plant association. *Bull. Torrey Bot. Club* 53 7–26. 10.2307/2479933 28247414

[B30] Gotelli N. J. Mccabe D. J. (2002). Species co-occurrence: a meta-analysis of J. M. Diamond’s assembly rules model. *Ecology* 83 2091–2096. 10.1890/0012-9658(2002)083[2091:SCOAMA]2.0.CO;2

[B31] Gotzenberger L. de Bello F. Brathen K. A. Davison J. Dubuis A. Guisan A. (2012). Ecological assembly rules in plant communities – approaches, patterns and prospects. *Biol. Rev.* 87 111–127. 10.1111/j.1469-185X.2011.00187.x 21692965

[B32] Graham C. S. Fine P. V. (2008). Phylogenetic beta diversity: linking ecological and evolutionary processes across space in time. *Ecol. Lett.* 11 1265–1277. 10.1111/j.1461-0248.2008.01256.x 19046358

[B33] Guo L. Q. Wang Q. H. Zhou H. C. Yang B. (1999). Main forest types in central Yunnan Plateau and transmulation tendency. *Yunnan For. Sci. Technol.* 1 25–38.

[B34] Han J. Shen Z. Ying L. Li G. Chen A. (2015). Early post-fire regeneration of a fire-prone subtropical *Pinus* mixed forest in Yunnan, southwest China: the effects of pre-fire vegetation, fire severity and topographic factors. *For. Ecol. Manage.* 356 31–40. 10.1016/j.foreco.2015.06.016

[B35] Han J. Ying L. Li G. Shen Z. (2016). Spatial patterns of species diversity in the herb layer of early post-fire regeneration in mixed *Pinus yunnanensis* forests. *Chin. J. Plant Ecol.* 40 200–211. 10.17521/cjpe.2015.0161

[B36] Hardy O. J. Sonke B. (2004). Spatial pattern analysis of tree species distribution in a tropical rain forest of Cameroon: assessing the role of limited dispersal and niche differentiation. *For. Ecol. Manage.* 197 191–202. 10.1016/j.foreco.2004.05.014

[B37] Harms K. E. Gagnon P. R. Passmore H. A. Myers J. A. Platt W. J. (2017). Groundcover community assembly in high-diversity pine savannas: seed arrival and fire-generated environmental filtering. *Ecosphere* 8:e01716 10.1002/ecs2.1716

[B38] He T. Lamont B. B. Downes K. S. (2011). Banksia born to burn. *New Phytol.* 191 184–196. 10.1111/j.1469-8137.2011.03663.x 21388378

[B39] Huang B. Zhao Y. Huo D. Su W. Zhang G. (2016). Temperature effect on cone-opening time and seed germination of *Pinus yunnanensis*. *Seeds* 35 19–21.

[B40] Jiang H. Q. (1980). Distribution features and zonal regularity of vegetation in Yunnan. *Acta Bot. Yun.* 1 1–23.

[B41] Jin Z. Z. Peng J. (1998). *Vegetation of Kunming.* Kunming: Yunnan Science and Technology Press.

[B42] Kappes H. Sundermann A. Haase P. (2010). High spatial variability biases the space-for-time approach in environmental monitoring. *Ecol. Indic.* 10 1202–1205. 10.1016/j.ecolind.2010.03.012

[B43] Kayes L. J. Anderson P. D. Puettmann K. J. (2010). Vegetation succession among and within structural layers following wildfire in managed forests. *J. Veg. Sci.* 21 233–247. 10.1111/j.1654-1103.2009.01136.x

[B44] Keddy P. A. (1992). Assembly and response rules: two goals for predictive community ecology. *J. Veg. Sci.* 3 157–164. 10.2307/3235676

[B45] Keeton W. S. Franklin J. F. (2004). Fire-related landform associations of remnant old-growth trees in the southern Washington Cascade Range. *Can. J. For. Res.* 34 2371–2381. 10.1139/x04-111

[B46] Kembel S. W. (2009). Disentangling niche and neutral influences on community assembly: assessing the performance of community phylogenetic structure tests. *Ecol. Lett.* 12 949–960. 10.1111/j.1461-0248.2009.01354.x 19702749

[B47] Kraft N. J. Comita L. S. Chase J. M. Sanders N. J. Swenson N. G. Crist T. O. (2011). Disentangling the drivers of β diversity along latitudinal and elevational gradients. *Science* 333 1755–1758. 10.1126/science.1208584 21940897

[B48] Legendre P. (2000). Comparison of permutation methods for the partial correlation and partial mantel tests. *J. Stat. Comput. Simul.* 67 37–73. 10.1080/00949650008812035

[B49] Legendre P. Legendre L. (1998). *Numerical Ecology.* New York, NY: Elsevier.

[B50] Leibold M. A. Holyoak M. Mouquet N. Amarasekare P. Chase J. M. Hoopes M. F. (2004). The metacommunity concept: a framework for multi-scale community ecology. *Ecol. Lett.* 7 601–613. 10.1111/j.1461-0248.2004.00608.x

[B51] Lentile L. B. Smith F. W. Shepperd W. D. (2006). Influence of topography and forest structure on patterns of mixed severity fire in ponderosa pine forests of the South Dakota Black Hills, USA. *Int. J. Wildland Fire* 15 557–566. 10.1071/WF05096

[B52] Liu J. Qian H. Jin Y. Wu C. Chen J. Yu S. (2016). Disentangling the drivers of taxonomic and phylogenetic beta diversities in disturbed and undisturbed subtropical forests. *Sci. Rep.* 6:35926. 10.1038/srep35926 27775021 PMC5075936

[B53] Lopez B. Burgio K. Carlucci M. Palmquist K. Parada A. Weinberger V. (2016). A new framework for inferring community assembly processes using phylogenetic information, relevant traits and environmental gradients. *One Ecosyst.* 1:e9501 10.3897/oneeco.1.e9501

[B54] Måren I. E. Kapfer J. Aarrestad P. A. Grytnes J. A. Vandvik V. (2018). Changing contributions of stochastic and deterministic processes in community assembly over a successional gradient. *Ecology* 99 148–157. 10.1002/ecy.2052 29065214

[B55] Murphy B. P. Lehmann C. E. R. Russell-Smith J. Lawes M. J. (2014). Fire regimes and woody biomass dynamics in Australian savannas. *J. Biogeogr.* 41 133–144. 10.1111/jbi.12204

[B56] Myers J. A. Chase J. M. Crandall R. M. Jimenez I. (2015). Disturbance alters beta-diversity but not the relative importance of community assembly mechanisms. *J. Ecol.* 103 1291–1299. 10.1111/1365-2745.12436

[B57] Myers J. A. Chase J. M. Jiménez I. Jørgensen P. M. Araujo-Murakami A. Paniagua-Zambrana N. (2013). Beta-diversity in temperate and tropical forests reflects dissimilar mechanisms of community assembly. *Ecol. Lett.* 16 151–157. 10.1111/ele.12021 23113954

[B58] Niu K. C. Liu Y. N. Shen Z. H. He F. L. Fang J. Y. (2009). Community assembly: the relative importance of neutral theory and niche theory. *Biodivers. Sci.* 17 579–593. 10.3724/SP.J.1003.2009.09142

[B59] O’Dwyer J. P. Lake J. K. Ostling A. Savage V. M. Green J. L. (2009). An integrative framework for stochastic, size-structured community assembly. *Proc. Natl. Acad. Sci. U.S.A.* 106 6170–6175. 10.1073/pnas.0813041106 19336583 PMC2663776

[B60] Pausas J. G. (2014). Bark thickness and fire regime. *Funct. Ecol.* 29 315–327. 10.1111/1365-2435.12372

[B61] Pellegrini A. F. A. (2016). Nutrient limitation in tropical savannas across multiple scales and mechanisms. *Ecology* 97 313–324. 10.1890/15-0869.1 27145607

[B62] Raevel V. Munoz F. Pons V. Renaux A. Martin A. Thompson J. D. (2012). Changing assembly processes during a primary succession of plant communities on Mediterranean roadcuts. *J. Plant Ecol.* 6 19–28. 10.1093/jpe/rts011

[B63] R Core Team (2011). *R: A Language and Environment for Statistical Computing.* Vienna: R Foundation for Statistical Computing Available at: http://www.r-project.org/

[B64] Scott A. C. Bowman D. M. J. S. Bond W. J. Pyne S. J. Alexander M. E. (2014). *Fire on Earth: An Introduction.* Oxford: Wiley Blackwell, 413.

[B65] Segre H. Ron R. De Malach N. Henkin Z. Mandel M. Kadmon R. (2014). Competitive exclusion, beta diversity, and deterministic vs. stochastic drivers of community assembly. *Ecol. Lett.* 17 1400–1408. 10.1111/ele.12343 25167950

[B66] Shen Z. H. Zhang X. S. Jin Y. X. (2000). An analysis of the topographical patterns of the chief woody species at dalaoling mountain in the three gorges region. *Acta Phytoecol. Sin.* 24 581–589.

[B67] Shryock D. F. Esque T. C. Chen F. C. (2015). Topography and climate are more important drivers of long-term, post-fire vegetation assembly than time-since-fire in the Sonoran Desert, US. *J. Veg. Sci.* 26 1134–1147. 10.1111/jvs.12324

[B68] Shugart H. H. (1984). *A Theory of Forest Dynamics.* New York, NY: Springer-Verlag 10.1007/978-1-4419-8748-8

[B69] Su W. Cui F. Zhao Y. Zhou R. Zhang G. Cao J. (2017). Canopy seed bank and serotinous cones of *Pinus yunnanensis* forests. *Acta Ecol. Sin.* 7 541–548.

[B70] Su W. Shi Z. Zhou R. Zhao Y. Zhang G. (2015). The role of fire in the central Yunnan Plateau ecosystem, southwestern China. *For. Ecol. Manage.* 356 22–30. 10.1016/j.foreco.2015.05.015

[B71] Tempel D. J. Gutiérrez R. J. Whitmore S. A. Reetz M. J. Stoelting R. E. Berigan W. J. (2014). Effects of forest management on California Spotted Owls: implications for reducing wildfire risk in fire-prone forests. *Ecol. Appl.* 24 2089–2106. 10.1890/13-2192.1 29188683

[B72] Thomaz S. M. Agostinho A. A. Gomes L. C. Silveira M. J. Rejmánek M. (2012). Using space -for-time substitution and time sequence approaches in invasion ecology. *Freshw. Biol.* 57 2401–2410. 10.1111/fwb.12005

[B73] Tuomisto H. (2010). A diversity of beta diversities: straightening up a concept gone awry. Part 2. Quantifying beta diversity and related phenomena. *Ecography* 33 23–45. 10.1111/j.1600-0587.2009.06148.x

[B74] Turner M. G. Baker W. L. Peterson C. J. Peet R. (1998). Factors influencing succession: lessons from large, infrequent natural disturbances. *Ecosystems* 1 511–523. 10.1007/s100219900047

[B75] Ulrich W. Zaplata M. K. Winter S. Schaaf W. Fischer A. Soliveres S. (2016). Species interactions and random dispersal rather than habitat filtering drive community assembly during early plant succession. *Oikos* 125 698–707. 10.1111/oik.02658

[B76] Vanschoenwinkel B. Buschke F. Brendonck L. (2013). Disturbance regime alters the impact of dispersal on alpha and beta diversity in a natural metacommunity. *Ecology* 94 2547–2557. 10.1890/12-1576.1 24400506

[B77] Watt A. S. (1947). Pattern and process in the plant community. *J. Ecol.* 35 1–22. 10.2307/2256497

[B78] West D. C. Shugart H. H. Botkin D. B. (eds). (1981). *Forest Succession: Concepts and Application.* New York, NY: Springer-Verlag 10.1007/978-1-4612-5950-3

[B79] Wu Z. He H. S. Liang Y. Cai L. Lewis B. J. (2013). Determining relative contributions of vegetation and topography to burn severity from LANDSAT imagery. *Environ. Manage.* 52 821–836. 10.1007/s00267-013-0128-3 23887487

[B80] Ying L. X. Han J. Du Y. S. Shen Z. H. (2018). Forest fire characteristics in China: spatial patterns and determinants with thresholds. *For. Ecol. Manage.* 424 345–354. 10.1016/j.foreco.2018.05.020

[B81] Zhang W. J. (2014). Research advances in theories and methods of community assembly and succession. *Environ. Skeptics Crit.* 3 52–60.

[B82] Zhang Y. T. Duan X. Li J. F. (1994). The partition in the forest conflagration and climate in the middle of Yunnan Province. *J. Southwest. For. Coll.* 14 172–176.

