# Supplementary figures and images for: Beta Diversity Patterns of Post-fire Forests in Central Yunnan Plateau, Southwest China: Disturbances Intensify the Priority Effect in the Community Assembly
Source: Front Plant Sci. 2018 Jul 11;9:1000. doi: 10.3389/fpls.2018.01000 (PMC6050402; doi:10.3389/fpls.2018.01000)

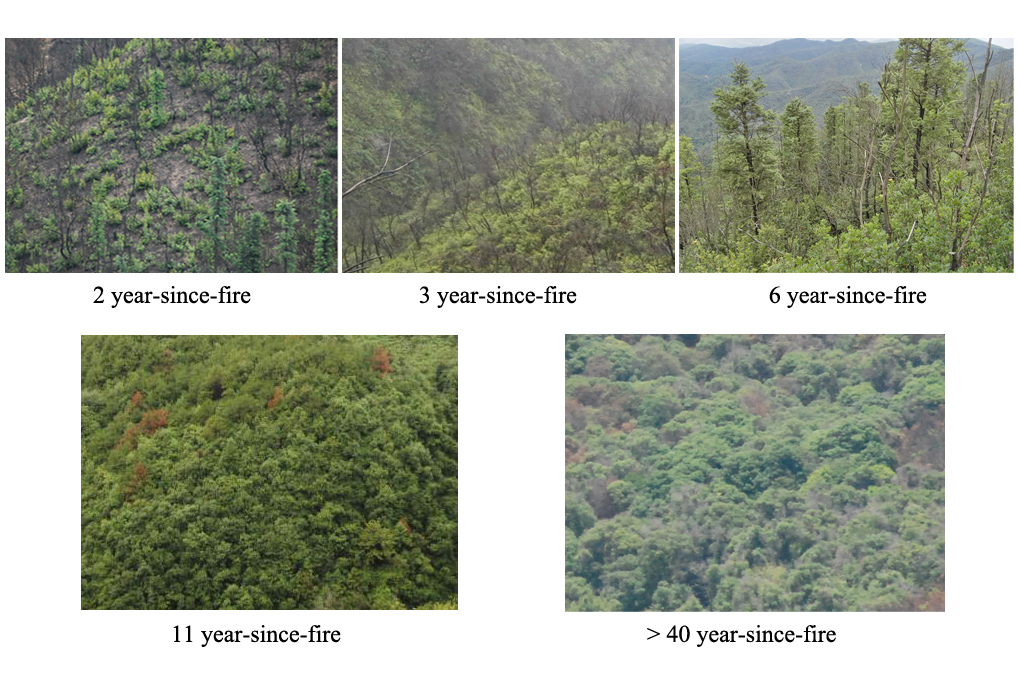

Supplement: FIGURE S1 — The appearance of post-fire regeneration forests of different year-since-fires. [file Image_1.JPEG]

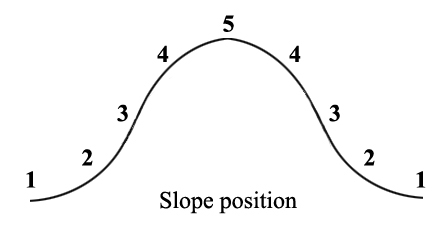

Supplement: FIGURE S2 — A diagram indicating slope positions of a vertical topographic profile. [file Image_2.JPEG]
